# EGF-Receptor against Amphiregulin (AREG) Influences Costimulatory Molecules on Monocytes and T Cells and Modulates T-Cell Responses

**DOI:** 10.1155/2023/8883045

**Published:** 2023-11-24

**Authors:** Stephan Dreschers, Christopher Platen, Louise Oppermann, Caitlin Doughty, Andreas Ludwig, Aaron Babendreyer, Thorsten W. Orlikowsky

**Affiliations:** ^1^Department of Neonatology, University Children's Hospital, Aachen, Germany; ^2^Institute of Pharmacology and Toxicology, Medical Faculty, RWTH Aachen University, Aachen, Germany

## Abstract

Amphiregulin (AREG) is a ligand of the epidermal growth factor receptor (EGFR) and has been shown to regulate the phagocytosis-induced cell death of monocytes in peripheral blood. AREG-dependent apoptotic signaling engages factors of the intrinsic and extrinsic apoptotic pathway, such as BCL-2, BCL-XL, and death ligand/receptor CD95/CD95L. Here, we tested the hypothesis that AREG influences costimulatory monocyte functions, which are crucial for T-cell responses. We found a stronger expression of AREG and EGFR in monocytes compared to lymphocytes. As a novel function of AREG, we observed reduced T-cell proliferation following polyclonal T-cell stimulation with OKT3. This reduction of proliferation occurred in the presence of monocytes as well as in their absence, monocyte signaling being replaced by crosslinking of OKT3. Increasing concentrations of AREG down-modulated the concentration of costimulatory B7 molecules (CD80/CD86) and HLA-DR on monocytes. In proliferation assays, CD28 expression on T cells was down-modulated on the application of OKT3 but unaltered by AREG. LcK activation, following OKT3-stimulation, was reduced in T cells that had been coincubated with AREG. The effects of AREG on T-cell phenotypes were also present when monocytes were depleted and OKT3 was crosslinked. The rearranged expression of immunological synapse proteins was accompanied by an alteration of T-cell polarization. Although the proportion of regulatory T cells was not shifted by AREG, IL-17-expressing T cells were significantly enhanced, with a bias toward TH1-polarization. Taken together, these results suggest that AREG acts as an immunoregulatory molecule at the interface between antigen-presenting cells and T cells.

## 1. Introduction

Upon bacterial infection, the orchestration of defense and healing processes is essential for survival and a good outcome and for preventing organ failures and chronic illness. The naïve immune system of neonates is frequently overburdened with this multiplicity of challenges, which explains the vulnerability of newborns, causing a high number of fatalities. At the interface between acute host defense against microorganisms and T-cell responses, monocytes are crucial.

Amphiregulin (AREG) is a member of the epidermal growth factor (EGF) family and translates into a trans-membrane protein that can act juxtacrine via its receptor EGFR. Previously, we have shown that AREG is secreted differently in neonates and adults and that it influences monocyte survival in the context of bacterial infection [1].

Numerous cell types are capable of secreting AREG. Reportedly, CD4+ -lymphocytes naturally exhibit a higher activity of metalloproteases such as TACE (ADAM17), which are required for the shedding of AREG from the plasma-membrane. Regulatory T cells (T-regs) were found to be a source of AREG under IL-33 stimulation [2]. Other studies indicate that APCs, such as dendritic cells (DCs) and monocytes, produce and secrete AREG [1, 3, 4].

AREG's receptor, EGFR, also binds ligands such as EGF, TGF-*α*, betacellulin, and epiregulin [5](Sunaga N, 2015). AREG, however, differs from those ligands in having a lower specificity for the EGFR. Furthermore, AREG–EGFR complexes remain at the plasma-membrane, whereas the other ligand/receptor complexes are internalized and transported to the endolysosome. There, the signaling is interrupted via proteolysis of the complex. In contrast, the AREG/EGFR complex remains active for longer [6].

Until now, our group has addressed the question of whether and, if so, how AREG enables initiation of PICD (phagocytosis-induced cell death) using cord blood monocytes and monocytes from adults. We found AREG signaling to be interlocked with the activity of metalloproteases MMP-2, MMP-9, and TACE (ADAM17), subsequently leading to overexpression of BCL-2 family proteins and the shedding of CD95L to suppress PICD. In turn, through blockage of overactive MMPs on the plasma-membrane of cord blood monocytes, CD95L remains capable of initiating PICD [1].

T-cell activation and deletion are influenced by various receptors on accessory cells. A factor crucial for this process is the phosphorylated protein LcK (pLcK56), which belongs to the Src family kinases. In turn, the targets of LcK phosphorylation include coreceptors such as CD28 and CTLA4 and immune cell-specific adaptor proteins such as LAT, Zap70, and SLP-76, which act to integrate signals proximal to surface receptors. The interaction of CD4 and CD8 as coreceptors of the T-cell receptor with LcK initiates a tyrosine phosphorylation cascade, leading to T-cell responses [7].

Monocytes possess ligands for costimulatory or apoptosis-inducing T-cell molecules. As professional antigen-presenting cells, monocytes express molecules of the B7 family (CD80, B7-1, and CD86, B7-2), which stimulate T-cell CD28 family molecules and facilitate an effective immune response.

CD28 is widely recognized as the major costimulation pathway for naive T-cell activation, and the CD28/B7 pathway plays a central role in immune responses against pathogens, autoimmune diseases, and graft rejection (reviewed in the study of Kim and Choi [8]). CTLA-4 (cytotoxic T-lymphocyte-associated protein 4), also known as CD152, is a protein receptor that functions as an immune checkpoint and downregulates immune responses. CTLA-4 is constitutively expressed in T-regs but only upregulated in conventional T cells after their activation [9, 10]. CD80 and CD86 on antigen-presenting cells, e.g., monocytes, signaling through CD28 and possibly other unidentified receptors, are required for optimal immune surveillance. Costimulation through the CD80/CD86-CD28 pathway is a key event in the induction of both humoral and cellular immune responses (reviewed in the study of Kremer [11]).

Here, we set out to test the hypotheses that AREG-dependent signaling regulates interacting molecules on the monocytes and lymphocytes required for T-cell activation and proliferation and that AREG-stimulated T cells are capable of responding with TH1 or TH2 reactions. To this end, we investigated whether AREG expression was engaged in the architecture of the immunological synapse, giving rise to stimulatory or blocking effects on the proliferation and polarization of T cells.

## 2. Material and Methods

### 2.1. Patient Samples

The experimental procedure presented here was approved by the Ethics Committee of Aachen University Hospital (Permission No: EK150/09, October 6, 2009). All adult participants gave written informed consent prior to having venous blood samples taken. Only neonates delivered spontaneously and showing no signs of infection were accepted for this study. Health status was determined by examination of white blood cell count, interleukin-6, C-reactive protein, and clinical status. Immediately after cord ligation, umbilical cord blood samples were placed in heparin-coated tubes (10 IU/ml blood). Neither mothers in whom the amnion had become infected, or labor had been prolonged (>12 hr) nor SGA (small for gestational age) neonates nor preterm infants before 36 weeks of gestation were accepted for the study.

### 2.2. Mononuclear Cell Culture

Human cord blood mononuclear cells (CBMC), as well as human peripheral blood mononuclear cells (PBMC), were isolated using Ficoll density gradient centrifugation (Amersham, Freiburg, Germany). Afterward, the cells were washed with phosphate-buffered saline (PBS), and monocytes were separated from the remaining cell types using the magnetic cell sorting monocyte isolation kit II (Miltenyi Biotec, Bergisch Gladbach, Germany) according to the manufacturer's recommendations. Detected by flow cytometry, the method routinely yielded 95% purity of the population, while 90% CD14 positive cells were defined as the minimal cutoff value. The cells were cultivated by a standard procedure in VLE RPMI-1640 medium (Biochrom, Berlin, Germany) containing 10% heat-inactivated fetal bovine serum (FBS, Biochrom, Germany) and 1% Penicillin/Streptomycin (Thermo Fischer, Massachusetts, USA). Postphagocytic reaction experiments were performed in 24-well cell culture plates (Costar, Bodenheim, Germany) containing 1 × 10^6^ monocytes/ml.

### 2.3. Reagents and Antibodies

IFNy was purchased from PAN-biotech. For immune-characterization, we used anti-CD14 (clone MEM18; Immunotools, Frisoythe, Germany), anti-CD16 (BD Biosciences, Heidelberg, Germany), anti-CD80 (clone 2D10.4, Thermo Fisher, Waltham, MA, USA), anti-CD86 (clone It2.2; Thermo Fisher, Waltham, MA, USA), anti-HLA-DR (bd-biosciences), anti-CD3 (BD Biosciences, Heidelberg, Germany), anti-CD4 (BD Biosciences, Heidelberg, Germany), anti-CD8 (clone SK1, BD Biosciences, Heidelberg, Germany), anti-KI67 (Thermo Fisher, Waltham, MA, USA), anti-CD25 (Thermo Fisher, Waltham, MA, USA), anti-FoxP3 (Thermo Fisher, Waltham, MA, USA), anti-IL10 (Thermo Fisher, Waltham, MA, USA), anti-IL13 (clone N49-653; Thermo Fisher, Waltham, MA, USA), anti-IL17 (Thermo Fisher, Waltham, MA, USA), anti-AREG (R&D Systems, Minneapolis, MN, USA), anti-LcK antibody (clone BP-1, Santa Cruz. Texas, USA), anti-phospho-tyrosine (residue 505) LcK antibody (Cellsignalling Technology, Leiden, the Netherlands) in concentrations recommended by the supplier.

### 2.4. Stimulation and Depletion of Monocytes

For monocyte stimulation, recombinant human AREG obtained from R&D Systems (Minneapolis, USA) was aliquoted in sterile PBS with 0.1% BSA and then used at a final concentration of 0.5 or 0.05 *μ*g/ml. For comparison, monocytes were stimulated with human recombinant 0.1 *µ*g/ml EGF (Sigma, Taufkirchen. Germany).

Monocytes were removed by magnetic-cell-sorting (Myltenyi Biotech, Bergisch Gladbach, Germany). According to the manufacturer`s recommendations, PBMC were mixed with CD14-positive microbeads and run through LS columns. The flow-through was tested for purity. Samples with a monocyte population below 0.5% were stimulated with *α*-CD3/CD28 (see below).

### 2.5. Proliferation Assays

Mononuclear cell cultures were obtained from cord blood and adult healthy volunteers and stained with carboxyfluorescein succinimidyl ester (CFSE), according to the manufacturer's protocol (Invitrogen, Carlsbad, CA, USA). PBMCs and CBMCs were stimulated with 1 *μ*g/ml muromonab-CD3 (OKT3) (Janssen Cilag, High Wycombe, UK). The cell culture was supplemented with 10% heat-inactivated human serum, 2 mM glutamine, 100 IU/ml penicillin, and 100 mg/ml streptomycin. After 96 hr of incubation in a humidified atmosphere at 37°C and 5% CO_2_, diminished CFSE fluorescence intensity was analyzed by flow cytometry to determine polyclonal T-cell proliferation.

We selected two experimental approaches to study the role of monocytes in T-cell proliferation. PBMNCs were plated on culture dishes for 2 hr under standard conditions. Afterward, the supernatant was removed and put through to two further rounds of culture dish cultivation. With this method, the percentage of Mo was reduced to below 5%. Second, PBMNCs were subjected to MACS-based depletion of Mo using the human Pan Monocyte Isolation Kit (Mylteni Biotec, Bergisch Gladbach, Germany). The purified leucocyte fraction was used for further experiments. When indicated (Figure S1(e)), syngenic nontouched Mo was retitrated to lymphocytes to a percentage of 15%.

Additionally, some experiments were conducted with plate-coated and soluble OKT3 where indicated. In the case of OKT3/ *α*-CD28 costimulation, OKT3 was coated to the plate by 15 min preincubation in PBS. The concentration of *α*-CD28 was 10 *µ*g/ml.

### 2.6. Immunoprecipitation and Immunoblotting

For immunoprecipitation, 10^6^ PBMNC were lysed in radio-immunoprecipitation assay (RIPA) buffer (sodium chloride 150 mM, Tris–HCl 50 mM, Nonidet P40 1% w/v, sodium deoxycholate, 0.5% w/v, SDS 0.1% w/v, before use 10 ml of RIPA was supplemented with 10 *µ*l protease inhibitor cocktail (3755.1 from Roth, Germany)) at 4°C The lysate (250 *µ*l) was mixed to 5 *µ*l of anti-Lck antibody (Lck antibody clone BP-1 from Santa Cruz Biotechnology (Texas, USA) 200 *µ*g/ml) and incubated ON at 4°C. Afterward, 5 *µ*l Protein G Sepharose (193259, Abcam, Berlin, Germany) was added, and incubation continued for a further 2 hr at 4°C. After two subsequent cycles of washing in RIPA buffer, samples were subjected to gel electrophoresis and immunoblotting. Tyrosine-phosphorylation was detected by utilizing the anti-phospho-tyrosine antibody 4G10.

For immunoblot analysis, 6 × 10^6^ cells were subjected to SDS–PAGE, which was performed according to standard protocols. For imaging and quantification, a LAS 3000 imager (Fujifilm, Düsseldorf, Germany) combined with the Multi-Gauge software (Fujifilm, Düsseldorf, Germany) was used.

### 2.7. Statistical Analysis

GraphPad Prism 7 statistical software (GraphPad Software, La Jolla, USA) was used for all analyses. Data are presented as means + standard deviation and were statistically analyzed using either one-way analysis of variance (ANOVA) or two-way ANOVA with Bonferroni's multiple comparisons test. Values of *p*  < 0.05 were considered as statistically significant. Where indicated, a Student's *t*-test with Welch correction was performed.

## 3. Results

### 3.1. EGFR Expression and AREG Expression on Monocytes and T Cells

**Figure 1:**
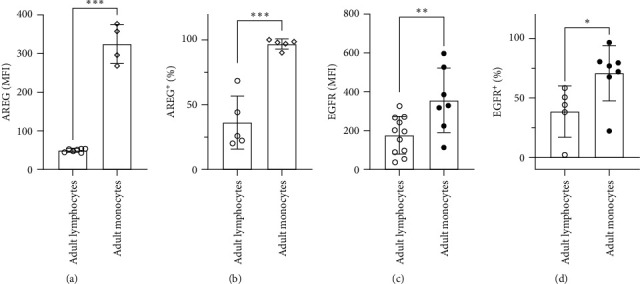
AREG and EGFR expression in monocytes and T cells. Percentage (a and c) of cells expressing intracellular AREG (a and b) and plasma-membrane located EGFR (c and d) without stimulation. The median expression was also assessed (b and d; columns represent groups indicated below, statistical analysis: Student's *t*-test,  ^*∗*^*p*  < 0.05,  ^*∗*^ ^*∗*^*p*  < 0.01,  ^*∗*^ ^*∗*^ ^*∗*^*p*  < 0.005).

Previous studies have revealed a stronger presentation of membrane-bound AREG (pro-AREG) on neonatal monocytes, derived from cord blood (CBMo) compared to monocytes from adult donors (PBMo) [1]. Here, we compared intracellular AREG levels in nonstimulated monocytes and lymphocytes from adult donors (Figures 1(a) and 1(b)). The intracellular AREG-concentration was found to be higher in Mo compared to lymphocytes. We also assessed the putative EGFR expression levels of the AREG ligand. Again, EGFR levels were elevated on monocytes compared to lymphocytes (Figures 1(c) and 1(d)).

### 3.2. Intrinsic AREG Stimulation Decreases Proliferation of T Cells

Since EGF signaling may affect cell activation, we analyzed T-cell proliferation in response to a polyclonal stimulus (OKT3) in the absence or presence of AREG. Stimulation of T-cell proliferation requires both crosslinking of CD3 (OKT3) molecules and costimulation of additional receptors located on the plasma-membrane of T cells [12].

**Figure 2:**
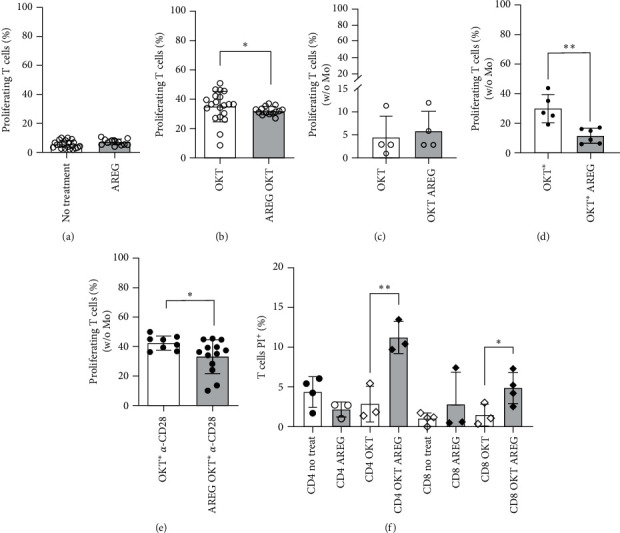
T-cell response upon stimulation with a polyclonal antibody (OKT3). Proliferation of T cells was determined by CFSE-assay. The proliferation rate was analyzed for PBMNC treated with AREG only (a). Proliferation in the presence of Mo was triggered by addition of OKT3 to PBMNC cultured as indicated (b). After removal of Mo (c and d), T-cell proliferation was induced with either soluble (c) or cross-linked OKT3 ( ^*∗*^; d and e) and CD28 (e), respectively. The percentage of apoptotic T cells was assessed by hypoploid DNA (Nicoletti) assay (f; statistical analysis for all charts: Student's *t*-test,  ^*∗*^*p*  < 0.05,  ^*∗*^ ^*∗*^*p*  < 0.01).

AREG did not cause proliferation of T cells in the absence of OKT3 mAb (Figure 2(a)). In the presence of Mo, OKT3-induced T-cell proliferation was significantly reduced by 12% 48 hr after the addition of AREG (Figure 2(b)). As a further control, we added soluble OKT3 to monocyte-depleted T cells and observed no proliferation (Figure 2(c)). Monocyte-depleted cell cultures supplied with crosslinked OKT3 and anti-CD28 mAb showed the same phenomenon with a comparable reduction of proliferation in the AREG-group, indicating that AREG may have a direct effect on the proliferative capacity of T cells (Figure 2(d)).

In order to clarify the necessity of monocytes in this context, we refined the experimental setup by comparing T-cell proliferation after the complete removal of monocytes with the MACS-based method. In detail, we compared (1) T cells from PBMC after monocyte depletion, crosslinking OKT3 and supplying anti-CD28 mAb as a costimulatory receptor with (2) T cells from PBMC after monocyte depletion with the addition of OKT3 mAb and anti-CD28 mAb noncrosslinked. All groups were tested in the absence or presence of the AREG stimulus (compare Figures 2(d) and 2(e); Figure S1(e)).

T cells cocultivated with Mo both with and without AREG stimulus showed proliferation after 48 and 72 hr incubation. However, at both time points, i.e., the F_1_ and F_2_ peaks, T cells showed decreased proliferation when cultivated with AREG. Evaluation of the histograms revealed that the decrease in T-cell proliferation in response to AREG stimulation is of statistical significance. The decrease in proliferation rate caused by AREG did not change between 48 and 72 hr (Figure S1(e)). In addition, we used EGF in one group as a high-affinity ligand to the EGFR. It should be noted that the addition of EGF did not lead to comparable effects regarding proliferation, either in a setting with OKT3 or in combination with anti-CD28.

We also investigated whether cell death can change the proportion of CD4+/CD8+ T cells (Figure 2(f)). Both CD4+ and CD8+ T cells underwent cell death after the addition of AREG to OKT3 stimulation (compare columns 3–4 and 7–8).

### 3.3. EGFR Stimulation Alters the Expression of Molecules at the Immunological Synapse

**Figure 3:**
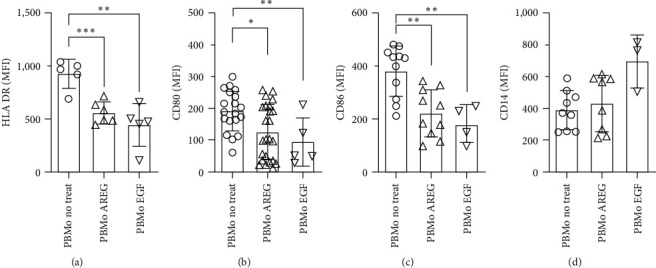
Regulation of surface molecules on Mo after addition of EGFR ligands. PBMC were cocultivated without or with AREG and EGF for 48 hr, and median densities of the indicated molecules assessed ((a) HLA-DR, (b) CD80, (c) CD86, (d) CD14; statistical analysis: Student's *t*-test,  ^*∗*^*p*  < 0.05,  ^*∗*^ ^*∗*^*p*  < 0.01,  ^*∗*^ ^*∗*^ ^*∗*^*p*  < 0.005).

Since T-cell proliferation depends on the costimulatory molecules of antigen-presenting cells [13], we analyzed several receptors on monocytes. A key molecule on these cells is the human leukocyte-antigen DR (HLA-DR), which is responsible for antigen presentation to T cells. In general, HLA-DR levels are described as being lowered during stressful situations, e.g., during sepsis. We and others also found a profound reduction and down-regulation of HLA-DR in neonates [14]. Here, we observed that HLA-DR was downregulated upon the addition of EGFR ligands AREG and EGF in PBMo (Figure 3(a)).

Next, the influence of AREG on costimulatory molecules CD80 and CD86 on monocytes, which activate lymphocytes and crucially trigger the immune response (reviewed in the study of Bolandi et al. [15]), was analyzed. We found that both cell surface markers were decreased dose-dependently in response to AREG stimulation. Addition of EGF had a comparable effect (Figure 3(b) and 3(c)). This was a selective decrease, since other markers, such as the CD14 molecule, were not sensitive to EGFR ligands. Furthermore, AREG did not cause monocyte cell death (Figure 3(d)).

**Figure 4:**
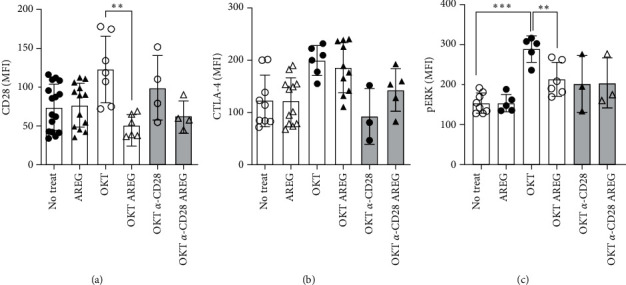
B7 receptors CTLA-4 and CD28 are responding differently to AREG. CD28 (a) and CTLA-4 (b) expression with or without the stimulation indicated. Intracellular levels of phosphorylated ERK were also assessed (c; statistical analysis: Student's *t*-test,  ^*∗*^ ^*∗*^*p*  < 0.01).

Since CD80/CD86 molecules may interact with CD28/CTLA-4 on the plasma-membrane of T cells, leading, among other factors, to an increased proliferation, we assessed their densities (Figure 4). In unstimulated T cells, CD28 and CTLA4-expression were not altered by AREG or EGF. In both OKT3-proliferating groups (monocytes present or monocytes absent, but crosslinked OKT3 plus anti-CD28 mAb), CD28 expression was significantly reduced in the presence of AREG (Figure 4(a)), while CTLA-4 expression was not affected (Figure 4(b)). Both CTLA-4 and CD28 can activate MAP-kinases such as ERK [16, 17]. We assessed the activation of ERK by detecting its phosphorylated form (Figure 4(c)). Stimulation of T cells with OKT3 increased ERK phosphorylation twofold. The addition of AREG reduced this phosphorylation to levels of unstimulated T cells. This effect could not be observed in unstimulated T cells treated with AREG. These findings indicate that both EGFR ligands are capable of reducing the densities of B7 molecules on monocytes but that regulation of CTLA-4 on proliferating adult T cells is restricted to AREG.

**Figure 5:**
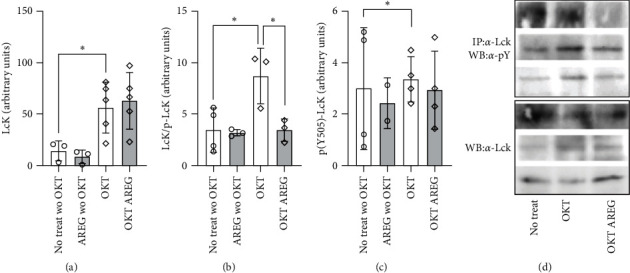
LcK tyrosine phosphorylation after stimulation with AREG and OKT3. LcK expression (a) and total tyrosine (Y) phosphorylation were detected via anti-LcK immunoprecipitation, as indicated (b). Phosphorylation of Y505 was also quantified (c). A representative immunoblot is also shown (d). Statistical analysis: Student's *t*-test,  ^*∗*^*p*  < 0.05,  ^*∗*^ ^*∗*^*p*  < 0.01.

In order to elucidate the downstream signaling of AREG on T cells, we precipitated Lck, a T-cell kinase signaling downstream of the CD3 complex. We first assessed and quantified the expression of LcK protein (Figure 5(a)). As expected, LcK concentrations were enhanced by OKT3 stimulation but not reduced by AREG. Next, the overall tyrosine phosphorylated form of LcK was immunoprecipitated (Figure 5(b)). Phospho-Lck was found to be present in lower concentrations in lysates from T cells stimulated with OKT3 and AREG compared to OKT3 stimulation only. This indicates that LcK activation is reduced by AREG (Figure 5(b)). To further characterize the role of LcK phosphorylation, we checked for the phosphorylation of Y residue 505, which was not changed by the addition of AREG (Figure 5(c)). Taken together, we obtained evidence that a reduced phosphorylation of LcK on Y-residues leads to a positive stimulation of downstream signaling.

### 3.4. AREG and Its Effects on T-Cell Polarization

**Figure 6:**
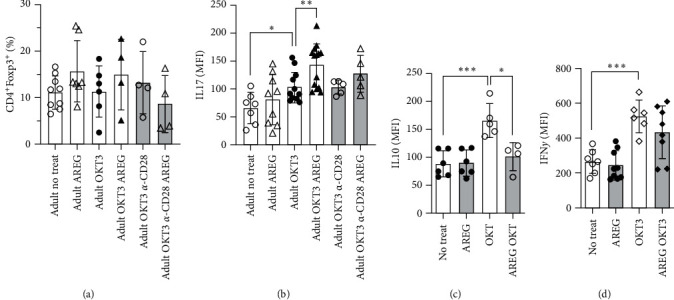
Dependance of T-cell polarisation on AREG. PBMNC were stimulated and cocultivated without or with AREG and EGF for 48 hr, as indicated ((a) CD4+/FoxP3+, (b) IL-17, (c) IL-10, (d) IFNy)). Percentages of FoxP3 positive cells and median densities of IL-17 were assessed ( ^*∗*^*p*  < 0.05,  ^*∗*^ ^*∗*^*p*  < 0.01).

Subsequently, based on a monitoring of the expression of cytokines and transcription factors (Figure 6), we selected FoxP3 and IL17 as prominent markers of T-cell polarization.

AREG had no significant effect on the expression of FoxP3 in T cells (Figure 6(a)). The cytokine IL-17 was enriched in OKT3-stimulated T cells while AREG synergistically enhanced IL-17 expression (Figure 6(b), compare columns 1, 4, 5). After the replacement of Mo by OKT3 and *α*-CD28, we could not observe any comparable effects, indicating that IL17 induction does not need Mo contact. In OKT3-stimulated T cells, both IL-10 and IFN-y content increased (Figures 6(c) and 6(d)). The addition of AREG to OKT3 could down-modulate IL-10 content but had no effect on IFN-y (Figures 6(c) and 6(d)).

Neonatal T cells have been described as reduced regarding T-cell blast formation [18]. We, therefore, addressed the question of whether AREG might play a role in this phenomenon (Figure S2). CBMo expressed significantly lower amounts of AREG (Figure S2(a)). Neonatal T cells expressed levels of AREG comparable to adult T cells. Furthermore, MDSC (myeloid-derived suppressor cells) expressed low levels of AREG. Neonatal T cells displayed a lower expression of CD28 after AREG and OKT3 costimulation. This effect was also observed when CBMo was mimicked by OKT3 *α*-CD28 stimulation in combination with AREG (Figure S2(b)). CTLA-4 expression was lower in T cells treated with AREG under cocultivation with OKT3 *α*-CD28 (Figure S2(c)). This pattern of costimulatory receptors was attributed to a significant but lower increase in proliferation after cocultivation with OKT3. However, AREG did not mitigate the already low proliferation (Figure S2(d)). In contrast, after the cocultivation of CBMNC with OKT3 and *α*-CD28, the proliferation rate was significantly stronger. The addition of AREG had no effect.

We show evidence that AREG acts directly on T cells, affecting their proliferation in the absence of monocytes, while acting as a modulator of Lck and Erk at the mono/T-cell interface when monocytes are present.

## 4. Discussion

In this manuscript, we provide evidence that AREG is involved in the modulation of the immunological synapse formed by Mo and T cells. This interface plays a functional role in the proliferation and polarization of T cells. We could show that Mo, as well as T cells, produced AREG and its receptor EGFR and that AREG downmodulated B7 molecules, resulting in antiproliferating effects (Figures 1– 3). This might be due to an increase of cell death (Figure 2). The phenomenon is partly independent of Mo and engages ERK signaling and LcK activation (Figures 2, 3, and 5). Second, AREG contributes to IL17 expression and a decrease in IL-10 production in T cells (Figure 6), supporting a pro-inflammatory surrounding. Taken together, AREG resets adult T cells to a state that has been observed in neonatal T cells.

T-cell activation and proliferation is a major response to infection. In recent years, our group has provided evidence that a hampering of T-cell activation and proliferative capacity causes an inappropriate immune response and promotes harmful sequelae and death. We found that the downregulation of B7 molecules and HLA-DR on neonatal monocytes leads to a reduced stimulation of T cells [19].

Molecules capable of modulating ligand/receptor interaction at the immunological synapse, therefore, present valuable candidates for immuno-modulation.

We have already shown that AREG acts via the EGFR/ERK2/AKT signaling axis and reduces the cell death of monocytes after challenge with *Escherichia coli* [1, 20]. Removal of subsets of monocytes may serve as a tool to control T-cell responses. Although it has been demonstrated that, in comparison to Mo, T cells express less AREG and EGFR (Figure 1), it cannot be ruled out that T cells can provide an autonomous, monocyte-independent AREG–EGFR signaling pathway. Due to their higher abundance compared to Mo, T cells can react on intrinsic AREG signaling.

Our results from proliferation assays demonstrate that this process is strongly dependent on Mo (Figure 2 and Figure S2). The proliferation-dampening effect of AREG can be explained, on the one hand, by its reduction of B7 molecules and HLA-DR (Figure 3) and, on the other hand, by the fact that AREG acts directly in *cis* on T cells. This is supported by our finding that CD28 is reduced on T-cell surfaces after the addition of AREG (Figure 4). CTLA-4 appears to play a minor role in this scenario. Furthermore, corresponding experiments with CBMNC showed a small but significant pro-proliferative effect on neonatal T cells. This effect is increased by costimulation with OKT3 and *α*CD28, which would support the notion of a Mo-dependent effect, as seen in adults. However, recent publications demonstrate a lack of B7 molecules and HLA-DR on CBMo [21, 22, 23]. The AREG-induced downregulation of CTLA-4 on neonatal T cells may outweigh the lack of stimulation by CBMo.

EGF and AREG compete for the same receptor, but only AREG diminishes the rate of proliferation (Figure 2 and Figures S1 and S2). In contrast, EGF, as well as AREG, induce similar effects with regard to the downregulation of HLA-DR, CD80, and CD86 on the Mo surface.

One might argue that EGF has only minor functions in septic events, but EGF secretion resulted in reduced mortality in an infection model [24]. Comparable effects were shown for other EGFR ligands, e.g., endoglin, HB-EGF, and FGF2 [25]. It is noteworthy that AREG, as well as other ligands of the EGFR, such as TGF-*α* and EGF, have the potential to induce phosphorylation in the tyrosine-kinase domain of the EGFR [26].

LcK becomes activated via *trans*-autophosphorylation. The phosphate group at position 505 is transferred to residue 394, leading to a conformational change that turns LcK into its active form [7]. We showed by immunoprecipitation that phosphorylated LcK is reduced after OKT3 AREG costimulation (Figure 5). Since the method chosen does not allow the investigator to discriminate between tyrosine 394 and 505, one might argue that this result provides no specific information concerning the activity state. However, since SHP-1 (Src homology region 2 domain-containing phosphatase-1) dephosphorylates and thereby inactivates LcK [27], the decrease in overall LcK dephosphorylation allows us to speculate that LcK dephosphorylation by SHP-1 is triggered by AREG. EGFR interacts with SHP-1, and AREG–EGFR ligation could activate SHP-1, which in turn deactivates LcK [28].

We selected two factors for studying T-cell polarization—the cytokine IL-17 and the transcription factor FoxP3 (Figure 6). IL17 served as a marker for TH17 polarization and FoxP3 expression for Treg polarization. Although these markers do not fully reflect the complicated processes in functional T cells, studies report that the proportion of Th17 to Treg can be used to distinguish between patients suffering from sepsis and septic shock [29]. Since no reaction was observable along the Treg polarization axis, the shift in Th17/Treg balance must have been driven by a reduction of IL17-producing T cells (Figure 6). This result could be interpreted as an acceptance of tolerance combined with the reduction of Th1 responses. One mechanism by which Th17/Treg balance can become skewed is through signaling of the AREG/EGFR axis, which changes the metabolic program of a T cell. This was shown in a study investigating the promotion of colorectal cancer [30].

We previously reported that AREG has anti-apoptotic properties in Mo [1]. Here, our experimental results provided evidence that AREG promotes cell death, at least of CD8+ T cells, without stimulation with OKT3 or OKT3/*α*-CD28 (Figure S2). In combination with OKT3, both CD4+ and CD8+ T cells were killed. However, OKT3 reduces CD4+ T cells after the addition of AREG in a setting mimicking the presence of Mo (Figure 2). When Mo are withdrawn, CD4+ T cells increase after the addition of AREG (Figure S2), thus underlining the importance of Mo during the proliferation of CD4+ T cells. Taken together, the balance of CD4+/ CD8+ T cells is shifted to a more prominent CD8+ population. A comparable scenario could be observed in AIDS patients [31].

The data collected in this study provides evidence that AREG is an immunomodulator, which acts on and via Mo to control T-cell proliferation and functional programing. Furthermore, we have shown that AREG can also act directly in T cells to further modulate the T-cell population via proliferation and cell death.

## Data Availability

Data are deposited in a repository and will be available after publication.

## Supplementary Materials

Supplementary Materials Figure S1: gating strategy and histogram analysis of proliferating T cells. Figure S2: AREG expression in neonatal and adult monocytes.
