# Supplementary material for: EGF-Receptor against Amphiregulin (AREG) Influences Costimulatory Molecules on Monocytes and T Cells and Modulates T-Cell Responses
Source: J Immunol Res. 2023 Nov 24;2023:8883045. doi: 10.1155/2023/8883045 (PMC10691888; doi:10.1155/2023/8883045)
Supplement: Supplementary Materials — Figure S1: gating strategy and histogram analysis of proliferating T cells. Figure S2: AREG expression in neonatal and adult monocytes. [file 8883045.f1.docx]

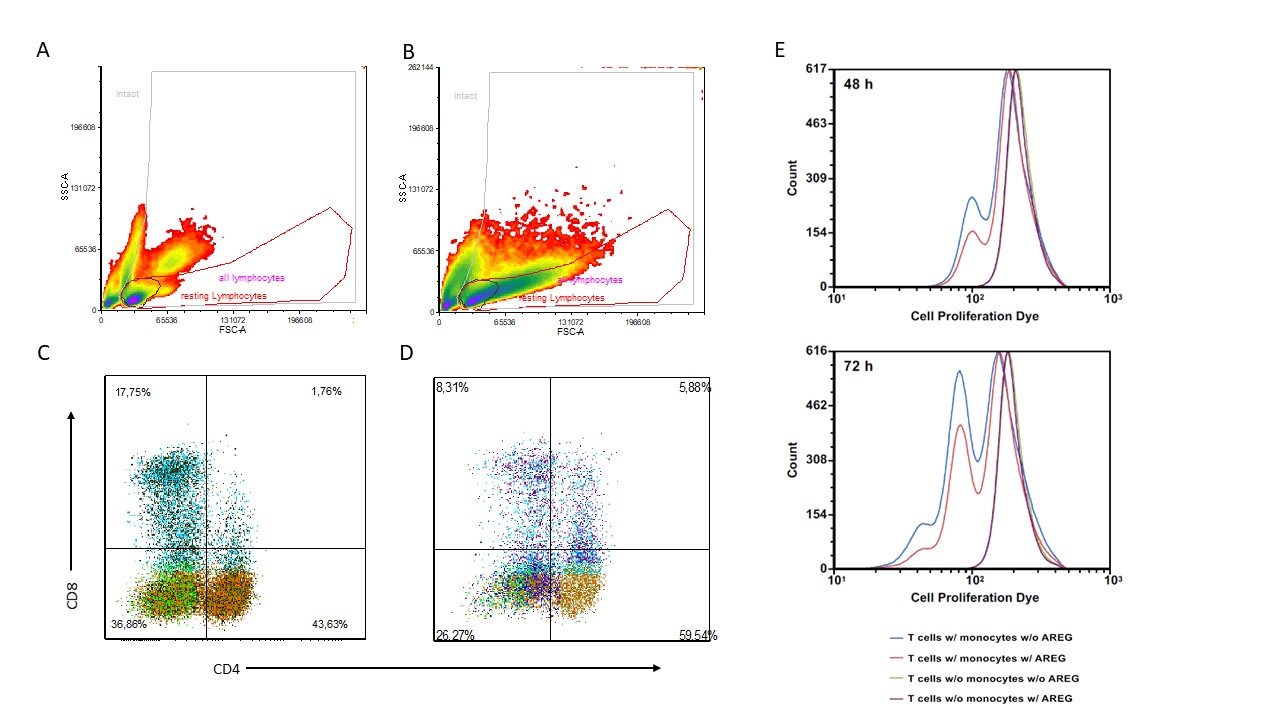

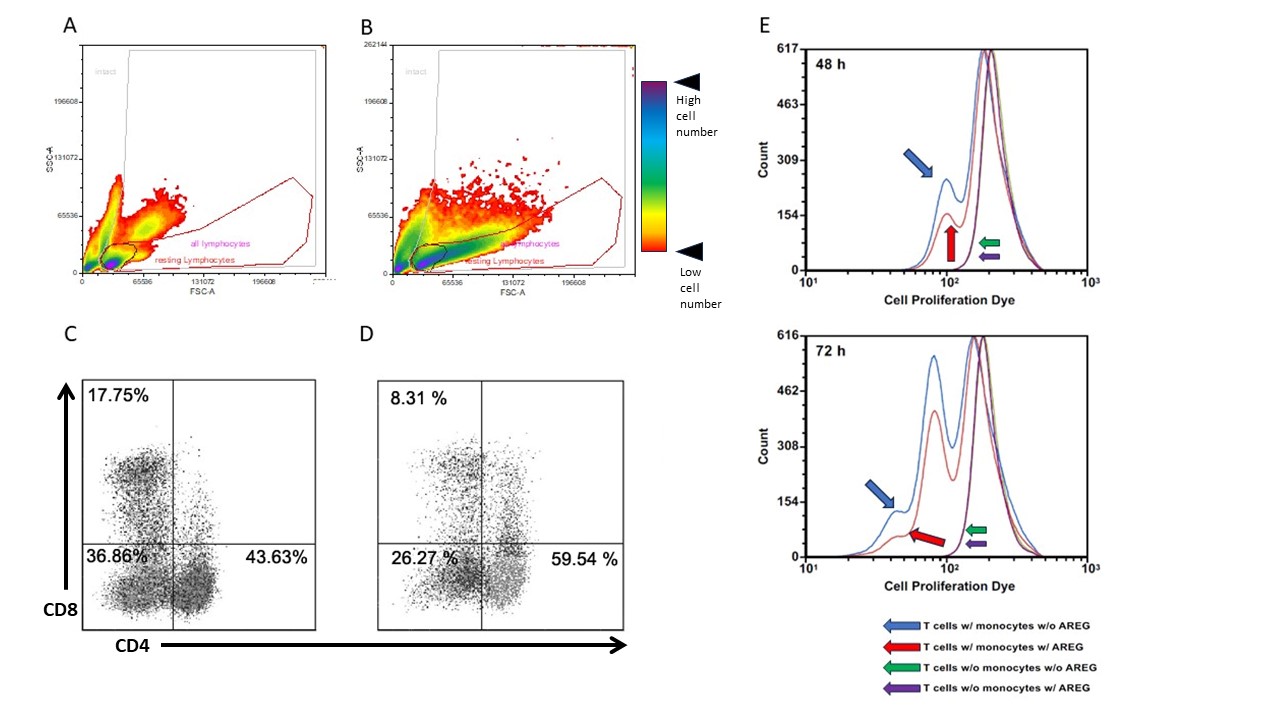
**Supplementary Figure 1:** **Gating strategy and histogram analysis of proliferating T cells.**

### Density Plots of PBMNC cultivated without (A) or with OKT3 (B) for 48 hrs. Heat map of cell densities is given beside. Gates for populations analyzed separately are designated in the density plots (A-B). Dot plots (C-D) show CD4^+^ and CD8^+^ positive T cells of the gate “resting” T cells (C) or “proliferating” T cells (D; “all lymphocytes” – “resting lymphocytes” = “proliferating lymphocytes”) of OKT3 stimulated PBMNC. Percentages of CD4^+^ and CD8^+^ Tcells are given in the quadrants. Typical histogram analysis of CFSE (cell proliferation dye) stained cells, treated as given below (E) and analyzed after indicated intervals. Blue and red arrows mark second and third generations of proliferating T cells.


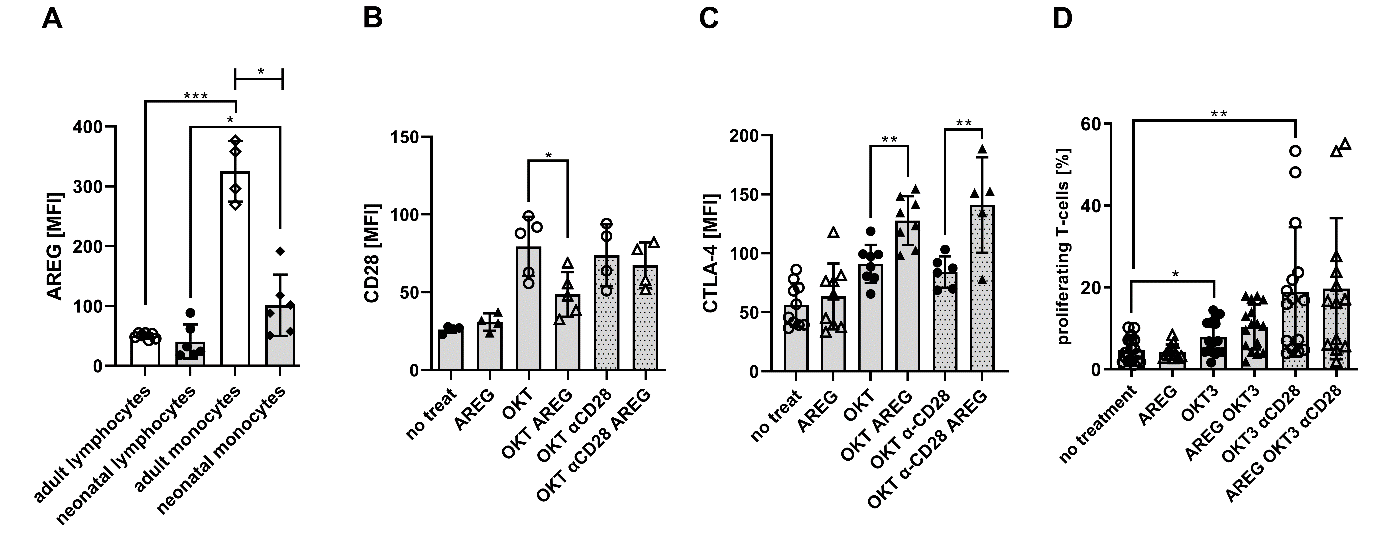


**Supplementary Figure 2: AREG expression in neonatal and adult monocytes.**

Intracellular AREG expression of adult (white bars) and neonatal Mo (grey bars, A). T cell receptor molecules CD28 (B) and CTLA-4 (C) was quantified and T cell proliferation (D) measured (blunt ended bar ANOVA* p<0.05; Student`s t-test, * p<0.05. ** p<0.01).
